# Surgical Treatment of Jacob's Disease: A Case Report Involving an Osteochondroma of the Coronoid Process

**DOI:** 10.1155/2013/253740

**Published:** 2013-06-13

**Authors:** Dale E. Stringer, Kourt B. Chatelain, Rahul Tandon

**Affiliations:** Department of Oral and Maxillofacial Surgery, Loma Linda University, Loma Linda, CA 92350, USA

## Abstract

Although it is one of the most common benign tumors of bone in the axial skeleton, the osteochondroma is relatively rare in the maxillofacial region. Its discovery on the coronoid process is even more rare. First described by Jacob in 1899, it remains a rare entity as only a few reported cases have been described in the literature. Nevertheless, the symptomatic features remain relatively nonspecific: limited opening, tightness, and slight expansion of the affected area with or without pain. The demographic features are more established, as it affects younger males. Definitive diagnosis is made after histological analysis, post-resection of the growth. We report a 27 year-old male with a history of limited opening and tightness of the mouth. Computed Tomography (CT) imaging revealed a well corticated exophytic protuberance from the left coronoid process. Left coronoidectomy and excision of the exophytic growth was performed, and was confirmed by histologic analysis to be an osteochondroma, demonstrating Jacob's disease.

## 1. Introduction

One of the most common benign tumors of bone is an osteochondroma, an osteocartilaginous exostosis. With its characteristic mushroom shape and cartilage-capped projection, it is a common tumor of the axial skeleton that is found most frequently in the metaphysis of the femur and tibia [1, 2]. Its path to formation follows bones that undergo endochondral ossification [3], which is supported by its prevalence in the described areas. Due to this developmental dependence, its occurrence in the maxillofacial region is relatively low since the majority of facial bones undergo intramembranous ossification [1]. In spite of this extremely low occurrence, osteochondromas can occur on the coronoid process and the condyle. 

Jacob's disease is a benign skeletal tumor referring specifically to the formation of an osteochondroma of the mandibular coronoid process. While this tumor is relatively rare throughout the body, it is even more rare in the maxillofacial region and, specifically, on the coronoid process [4]. Although first reported by Langenbeck in 1853, it was not until 1899 when Jacob described its distinguishing characteristics: a pseudoarthrosis joint between the coronoid and the zygomatic arch due to the formation of an osteochondroma [5].

Whether unilateral or bilateral, a common sign is expansion of the zygoma/zygomatic arch [6], accompanied by tightness and limited mouth opening [1]. Surprisingly, pain is not a common symptom of patients [6, 7]. Although the etiology of the disease is debated, some authors point towards a genetic or endocrine cause [8], while others lean towards hyperactivity of the temporal muscle or disc displacement of the TMJ [9]. In spite of clinicians disagreeing on the cause, the demographics seem to be more established: predominantly young males, with an average age of 35 [10]. 

Regardless of etiology and demographics, the treatment of Jacob's disease remains coronoidectomy and excision of the tumor [11], with recurrence of the osteochondroma relatively rare. We present a case of Jacob's disease treated by coronoidectomy along with removal of attached osteochondroma. 

## 2. Case Report

**Figure 1:**
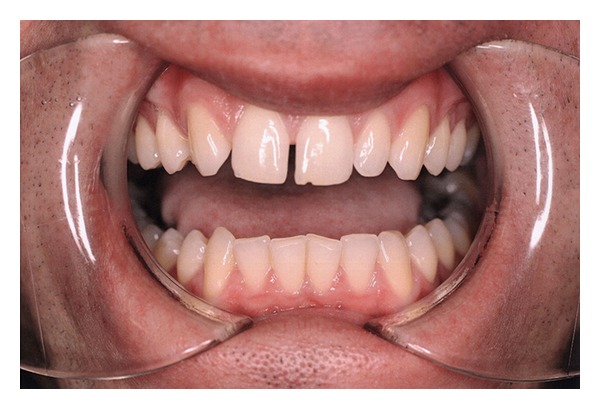
Physical examination reveals interincisal opening of 10 mm.

**Figure 2:**
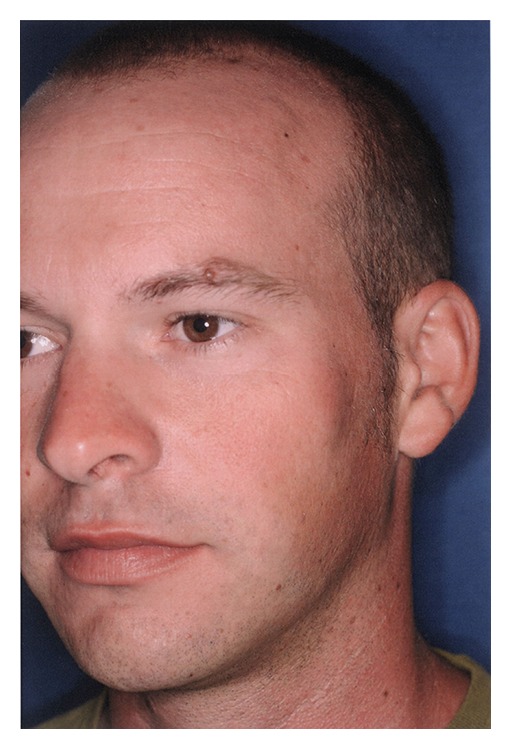
Left zygomatic arch expansion.

**Figure 3:**
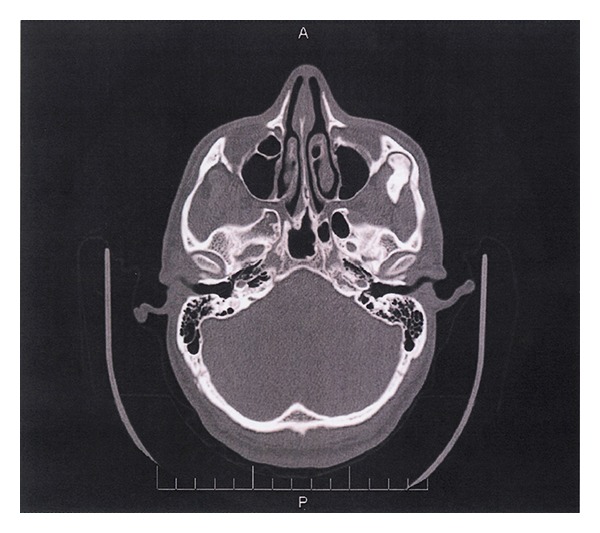
CT image of exophytic mass projecting from left coronoid process.

A 27-year-old male was referred for evaluation and possible surgical treatment due to limited opening on the left side. The patient had experienced some pain and asymmetry on his left side. Physical examination revealed an interincisal opening of 10 mm (Figure 1) and left zygomatic arch expansion (Figure 2). A panoramic radiograph showed that the left coronoid process was slightly elongated when compared with the contralateral side. A computed tomography (CT) scan revealed well-corticated exophytic protuberance projecting anteriorly and superiorly from the hypertrophied left coronoid process (Figure 3). The patient was then scheduled for left coronoidectomy and excision of the exophytic mass (osteochondroma). 

## 3. Description of Procedure

**Figure 4:**
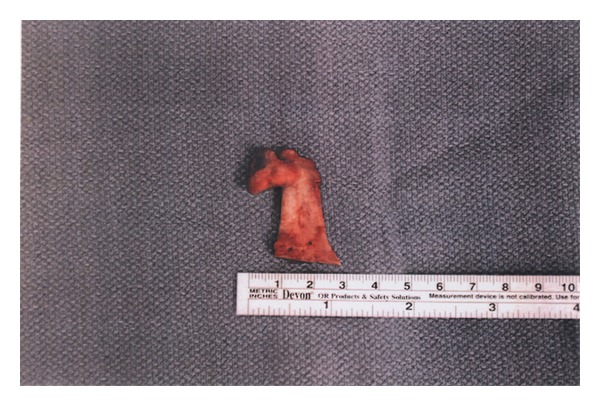
Resected osteochondroma and coronoid process.

The patient was taken to the operating room and, after fiberoptic nasal intubation, was prepped and draped for transoral incisions. The first incision was made with a Bovie electrocautery along the left ascending ramus down to the body of the mandible. Subperiosteal dissection was carried out superiorly to the sigmoid notch and lower portion of the coronoid. A Bauer retractor was placed in the sigmoid notch in order to allow for direct visualization of the coronoid process. With a reciprocating saw, the coronoid process was osteotomized. Careful dissection allowed for removal of the attachments of the inferior portion of the coronoid process. A second incision was then made in the left maxillary vestibule, and a full-thickness mucoperiosteal flap was raised exposing the buttress and zygomatic arch. The osteochondroma was palpated and, using blunt dissection, was released from its attachments to the zygoma down to the coronoid and the whole process removed together (Figure 4). 

**Figure 5:**
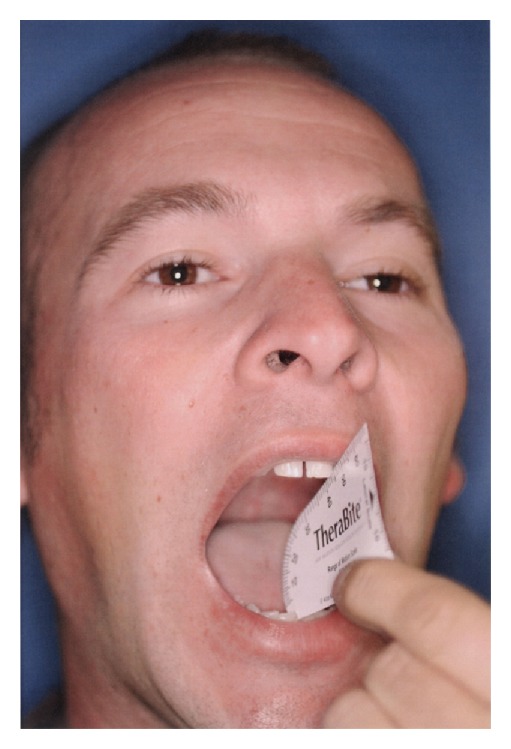
Postoperative interincisal opening of more than 40 mm.

Histopathologic analysis was consistent with the dense bone and associated portion of cartilage. Postoperatively, after physical therapy with Therabite, the patient was able to obtain an opening of greater than 40 mm (Figure 5). 

## 4. Discussion

Osteochondroma is a benign tumor of bone and cartilage that is more commonly seen in the axial skeleton than in the maxillofacial skeleton. This cartilage-capped mushroom-like growth is usually first noted during routine radiographic examination or during palpation of the affected area [1]. Any of these signs warrant computed tomography (CT) scan with three-dimensional reconstruction to confirm the clinician's suspicions. As first described in 1899, Jacob's disease can eventually lead to the formation of a pseudojoint between the zygoma and the coronoid process. While remaining relatively asymptomatic in its beginning stages, patients do complain of progressive limitation in opening of their mouth. These symptoms are followed or accompanied by tightness within the joint area and deviation toward the affected side during mouth opening [2]. This growth can result from many different etiologies such as trauma, chronic disc displacement, and genetic abnormalities [3]. Although the causative factor remains debatable, many believe that periosteal hyperactivity leads to ectopic formation of metaplastic cartilage [12]. This growth eventually moves laterally and forms a pseudojoint with the medial surface of the zygomatic arch [7]. Due to this anatomical closeness to the TMJ, the signs and symptoms can be mistaken for temporomandibular disorder (TMD), which could lead to mistreatment [13]. 

Although extraoral approaches have been advocated in the past [14, 15], we elected the more common intraoral technique. Radiographic images provided a rough estimate of the size of the lesion, which showed a lesion with the appropriate dimensions for the intraoral approach. Additionally, the excessive amounts of scarring and high risk for injury to surrounding neurovascular structures seen with the extraoral approach made the choice of an intraoral approach a more practical one. Although the intraoral approach eliminates many of these risks, there is a small chance of buccal fat pad herniation into the surgical site. However, this risk is relatively rare as long as the incision is not made too superiorly and medially. Our patient's follow-up showed that he was healing well and had improved mouth opening. We hope that our technique, which has been established for appropriately sized lesions, is a viable option for surgeons if faced with the rare case of an osteochondroma of the coronoid process.
